# Sequential development of hyperinsulinemic hypoglycemia and type 1 diabetes mellitus in a male child with trisomy 13

**DOI:** 10.1530/EDM-25-0001

**Published:** 2025-04-24

**Authors:** Satoru Sugimoto, Hidechika Morimoto, Tatsuji Hasegawa, Yasuhiro Kawabe

**Affiliations:** Department of Pediatrics, Graduate School of Medical Science, Kyoto Prefectural University of Medicine, Kyoto, Japan

**Keywords:** trisomy 13, Patau syndrome, hyperinsulinemic hypoglycemia, type 1 diabetes mellitus

## Abstract

**Summary:**

We present a rare case of a 19-month-old boy with trisomy 13 who initially presented with hyperinsulinemic hypoglycemia (HH) at 1 month of age and later developed type 1 diabetes mellitus (T1DM). While cases of HH or T1DM alone have been reported in trisomy 13 patients, this is the first known report of both conditions occurring sequentially in a single individual. No previous reports have described the sequential progression from HH to T1DM in any population, highlighting this as an unprecedented clinical observation. This case underscores the complex nature of metabolic disorders in trisomy 13 and provides insights into the underlying mechanisms linking this chromosomal anomaly to the development of both HH and T1DM.

**Learning points:**

- Sequential progression from HH to T1DM in both trisomy 13 and the general population is unprecedented.
- The coexistence and progression of HH and T1DM underscore the intricate and multifaceted nature of metabolic disorders in trisomy 13.
- Routine monitoring of blood glucose and C-peptide levels may facilitate the detection of metabolic transitions in patients with trisomy 13.

## Introduction

Trisomy 13, also known as Patau syndrome, is a rare chromosomal disorder associated with multiple congenital anomalies and severe intellectual disability (1). Although metabolic and endocrine abnormalities are occasionally reported in trisomy 13 patients, cases involving both hyperinsulinemic hypoglycemia (HH) and type 1 diabetes mellitus (T1DM) are exceedingly rare.

HH, characterized by excessive insulin production leading to persistent hypoglycemia, has been observed in some cases of trisomy 13 (2, 3). T1DM, an autoimmune disorder causing beta-cell destruction, is virtually unheard of in this population. To date, only a single case report has documented the coexistence of T1DM in a patient with trisomy 13 (4). However, the sequential progression from HH to T1DM in a single individual has not been documented in patients with trisomy 13 or any other population, emphasizing the novelty of this case and its contribution to the existing body of medical knowledge.

## Case presentation

### Neonatal course and diagnosis of HH

The patient was born at 38 weeks of gestation to a 43-year-old mother. Fetal ultrasonography revealed no abnormalities during the pregnancy. At birth, his weight was 2,854 g (+0.2 SD for Japanese standards) and his length was 47.5 cm (−0.3 SD for Japanese standards). The Apgar scores were 7 at 1 min and 8 at 5 min. Multiple congenital anomalies were noted at birth, including scalp defect, low-set ears, cleft palate and micropenis. Echocardiography revealed no structural heart abnormalities. Due to recurrent episodes of oxygen desaturation, the patient required endotracheal intubation and mechanical ventilation. On day 7 of life, G-band karyotyping was performed, confirming the diagnosis of trisomy 13 with a karyotype of 47,XY,+13.

After birth, the patient required glucose infusions to stabilize blood glucose levels. While nasogastric milk feeding was gradually increased, the maximum glucose infusion rate temporarily reached 6.4 mg/kg/min to maintain euglycemia. On day 23 of life, following extubation, the patient did not require reintubation; however, due to persistent respiratory instability, laryngofiberoscopy was performed, leading to a diagnosis of tracheomalacia. Subsequently, a tracheostomy was performed. Attempts to wean off glucose infusions on day 31 of life resulted in hypoglycemia, with a blood glucose level of 48 mg/dL. Blood samples obtained during hypoglycemia revealed findings consistent with HH, including a serum insulin level of 3.2 µIU/mL (reference range (RR): 1.84–12.20 µIU/mL), total ketone bodies of 34 μmol/L (RR: 0–130 μmol/L), acetoacetate at 13 μmol/L (RR: 0–55 μmol/L), 3-OH-butyric acid at 21 μmol/L (RR: 0–85 μmol/L) and non-esterified fatty acids of 219 µEq/L (RR: 140–850 µEq/L), indicating suppressed ketogenesis and lipolysis due to hyperinsulinism. The patient was started on diazoxide at a dose of 5 mg/kg/day, which effectively stabilized blood glucose levels. The intravenous line was withdrawn on day 59 of life after milk feeding was established. Following the discontinuation of intravenous therapy, blood glucose levels remained stable under the diazoxide treatment, and the patient was discharged on day 105 of life.

### Clinical course and diagnosis of T1DM

During outpatient follow-up, seven non-fasting blood glucose measurements were obtained at regular intervals under diazoxide therapy, ranging from 77 to 97 mg/dL. At 18-months of age, approximately 28 days before admission, the patient began to experience episodes of hyperglycemia. At that time, the diazoxide dose was 3.7 mg/kg/day. Blood glucose levels were consistently measured at >250 mg/dL. Fourteen days before admission, diazoxide therapy was discontinued due to persistent hyperglycemia; however, elevated glucose levels continued. Ten days before admission, the patient developed a fever and was evaluated at a nearby otolaryngology clinic, where he was diagnosed with acute otitis media. At that time, blood glucose levels were recorded at 300 mg/dL, indicating persistent hyperglycemia.

Upon admission to our hospital, the patient presented with signs of diabetic ketoacidosis (DKA), including weight loss of 5.0% over 2 weeks, with a current weight of 10.5 kg. Laboratory findings confirmed DKA, revealing severe hyperglycemia with a blood glucose level of 377 mg/dL, an elevated HbA1c of 8.7% and metabolic acidosis characterized by a blood pH of 7.278, bicarbonate (HCO3^−^) of 16.9 mmol/L, a base excess of −9.1 mmol/L and a lactate level of 2.0 mmol/L. Ketone body analysis showed markedly elevated total ketones (8,278 μmol/L), with acetoacetate at 1,676 μmol/L and 3-OH-butyric acid at 6,602 μmol/L. On subsequent days, serum autoantibody testing confirmed T1DM, with anti-GAD antibody >2,000 U/mL (RR: 0.0–4.9 U/mL) and anti-IA-2 antibody 14.0 U/mL (RR: 0.0–0.5 U/mL). Thyroid function tests indicated normal thyroid function. The patient’s hemoglobin F (HbF) level was 12.8%. In trisomy 13, persistent HbF elevation is often observed, which may result in HbA1c measurements not accurately reflecting glycemic trends depending on the measurement method used (4). This case’s HbA1c value of 8.7% reflects a measurement corrected for the elevated HbF level because our hospital’s measurement system automatically adjusts HbA1c for HbF interference.

Extracellular fluid dehydration was managed and intensive insulin therapy was initiated using a multiple daily injection regimen. The patient’s acute otitis media improved with antibiotic therapy. On the 8th day of hospitalization, the treatment was transitioned to sensor-augmented pump (SAP) therapy using the Medtronic MiniMed 770G system. The patient’s condition stabilized, and he was discharged on the 15th day of hospitalization.

### Outcome and follow-up

During outpatient follow-up, the patient continued SAP therapy. By 21 months of age, HbA1c levels decreased to approximately 7.0% and remained stable around this level until 27 months of age. The fasting C-peptide level, which was 0.96 ng/mL at the admission, progressively declined over time, reaching 0.03 ng/mL at 27 months of age, suggesting a diminished endogenous insulin secretion.

## Discussion

This report documents the first known case of sequential development of HH followed by T1DM in a patient with trisomy 13. A comprehensive review of the literature revealed no prior reports of HH transitioning into T1DM in any population, emphasizing the novelty of this case. While a 2021 case report described the coexistence of T1DM in a patient with trisomy 13 (4), it did not involve HH, distinguishing this case as unique.

HH is a rare metabolic condition characterized by excessive insulin secretion, resulting in recurrent hypoglycemia. To date, two published cases have documented HH in patients with trisomy 13, highlighting its potential association with this chromosomal abnormality (2, 3). One was classified as transient (2), while the other reported a case in which diazoxide treatment was tapered from 1.5 years of age, with no further episodes of hypoglycemia observed thereafter (3). Taken together with our case, HH associated with trisomy 13 does not necessarily align with the typical definition of transient hyperinsulinism, as it can persist beyond the neonatal period and may require prolonged diazoxide therapy. In addition, a single report has described the coexistence of T1DM and trisomy 13 (4). Although no evidence currently exists to suggest that chromosome 13 plays a direct role in the pathogenesis of either HH or T1DM, several genes located on chromosome 13 are known to influence insulin regulation and pancreatic function. For instance, CDX3 (caudal-type homeobox 3) has been implicated in the transcriptional regulation of insulin (5). PDX1 (pancreatic and duodenal homeobox 1) has been shown to play a critical role in maintaining pancreatic homeostasis. Mutations in PDX1 are associated with insulin-dependent diabetes mellitus (6, 7). The gene dosage effect caused by trisomy 13 may alter the normal function of these genes, potentially contributing to the development of these metabolic disorders.

To our knowledge, no prior case has documented the progression from HH to T1DM in trisomy 13 or any other population. Increasing evidence suggests that advancements in multidisciplinary medical care, including surgical interventions, have significantly improved survival rates among patients with trisomy 13 (8, 9). These advancements may allow for the identification of additional cases in which a single individual with trisomy 13 develops both HH and T1DM, thereby supporting the hypothesis that trisomy 13 fosters a pathological environment conducive to the coexistence of these two contrasting metabolic disorders.

## Conclusion

This report documents the first known case of sequential development of HH followed by T1DM in a patient with trisomy 13. The coexistence and progression of these metabolic conditions may reflect complex pathophysiology of trisomy 13. Routine monitoring of blood glucose and C-peptide levels may be useful in detecting metabolic transitions in patients with trisomy 13, and further studies are needed to elucidate the mechanisms driving these rare metabolic disorders.

## Declaration of interest

The authors declare that there is no conflict of interest that could be perceived as prejudicing the impartiality of the work reported.

## Funding

This study did not receive any funding from public, commercial or not-for-profit organizations.

## Patient consent

Informed written consent for the publication of its clinical details was obtained from the patient’s legal guardian.

## Author contribution statement

SS, HM, TH and YK were responsible for the clinical management of the patient and the collection of relevant medical data. SS drafted the initial manuscript and all authors contributed to its revision. YK conducted the literature review to support the discussion. All authors reviewed and approved the final manuscript for submission.
